# Supplementary figures and images for: Genome-wide identification and characterization of NAC genes in Brassica juncea var. tumida
Source: PeerJ. 2021 May 5;9:e11212. doi: 10.7717/peerj.11212 (PMC8106399; doi:10.7717/peerj.11212)

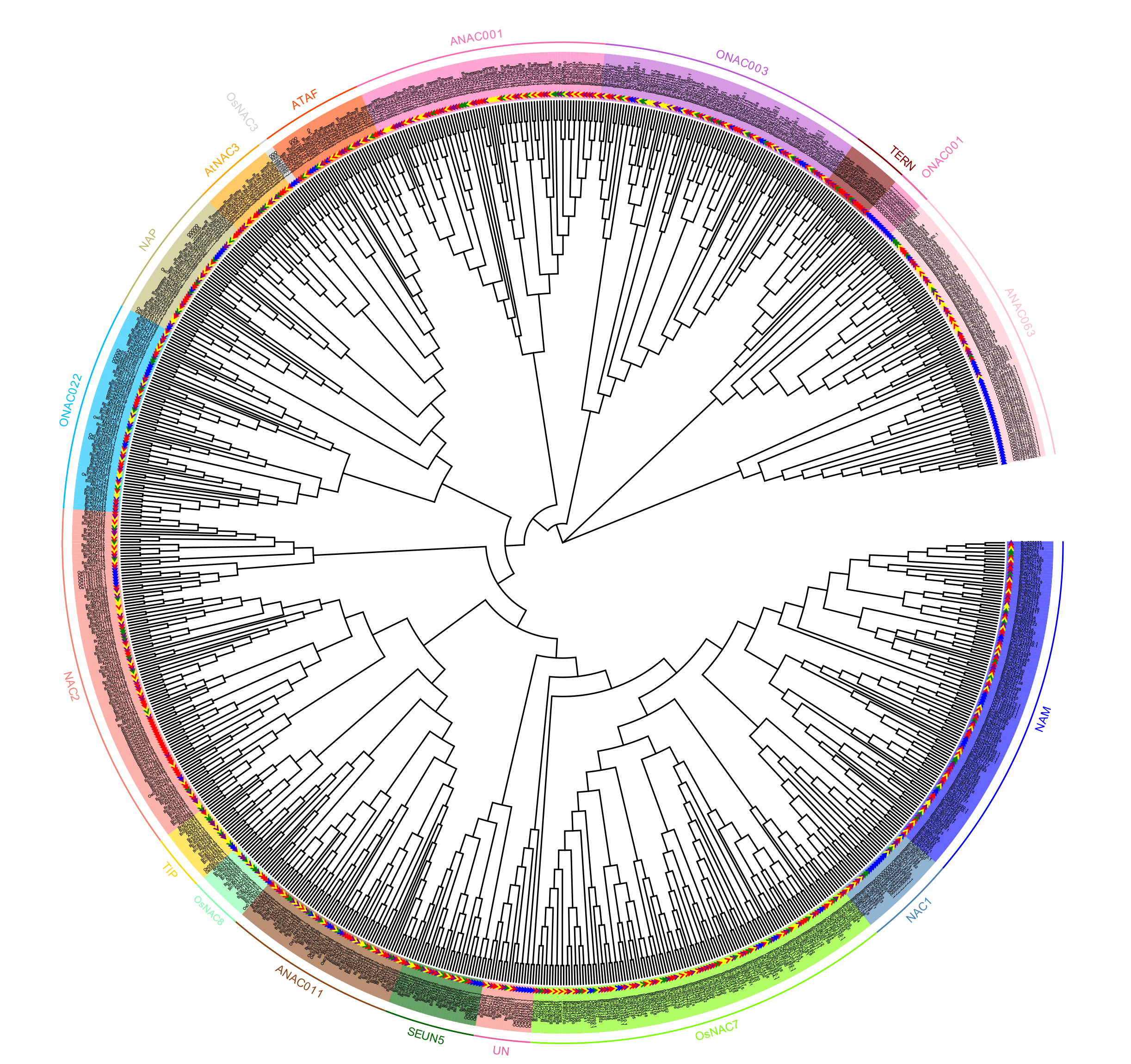

Supplement: Figure S1 — Multiple sequence alignment of full-length NAC proteins was done using muscle method and the phylogenetic tree was constructed using MEGAX by the neighbor-joining method with bootstrap 1000 replicates. [file peerj-09-11212-s003.png]

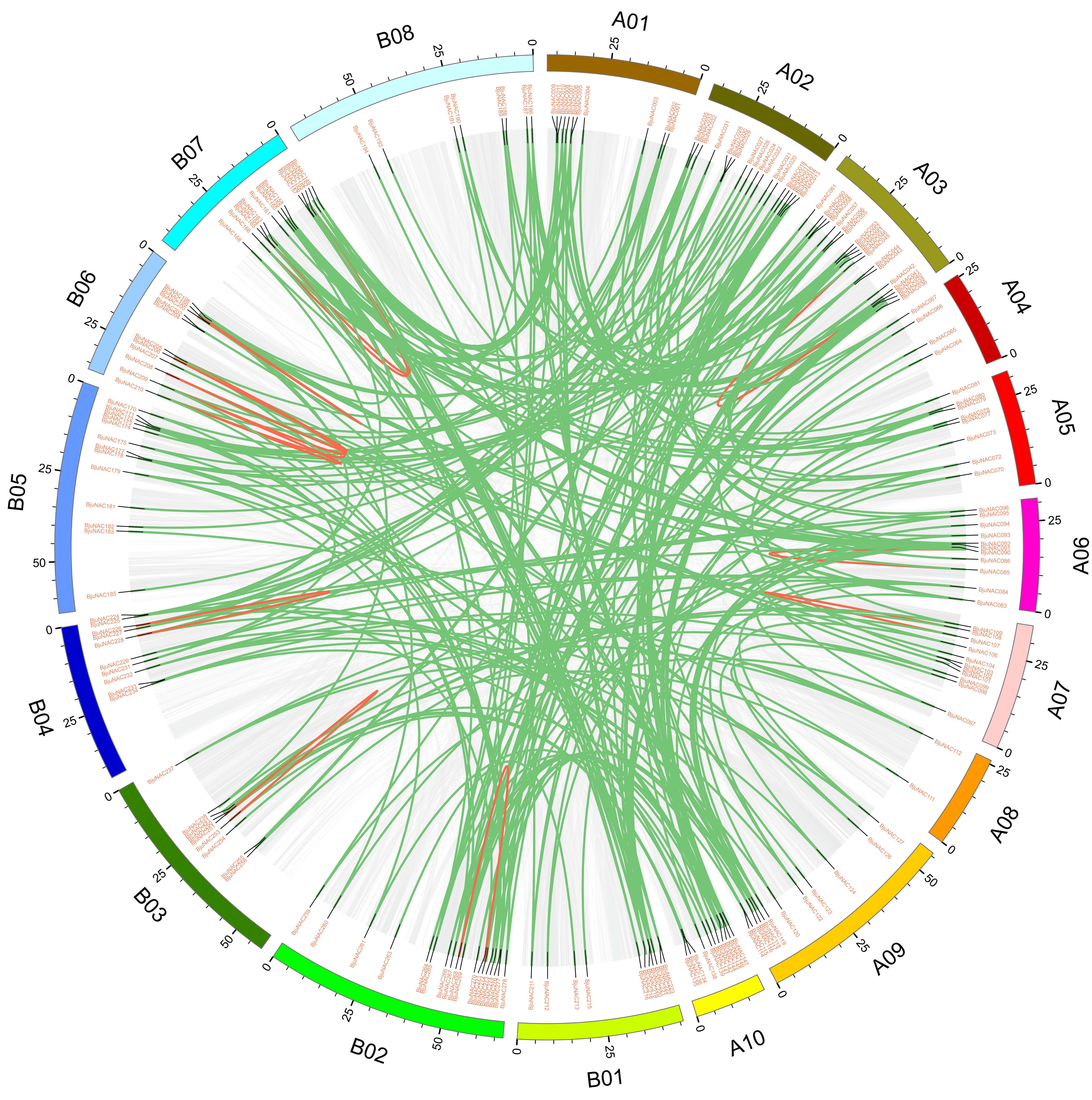

Supplement: Figure S2 — Collinear blocks represented by grey background. 288 BjuNAC duplication pairs are linked with green lines, and 15 tandem duplication pairs are linked with red lines. [file peerj-09-11212-s004.png]

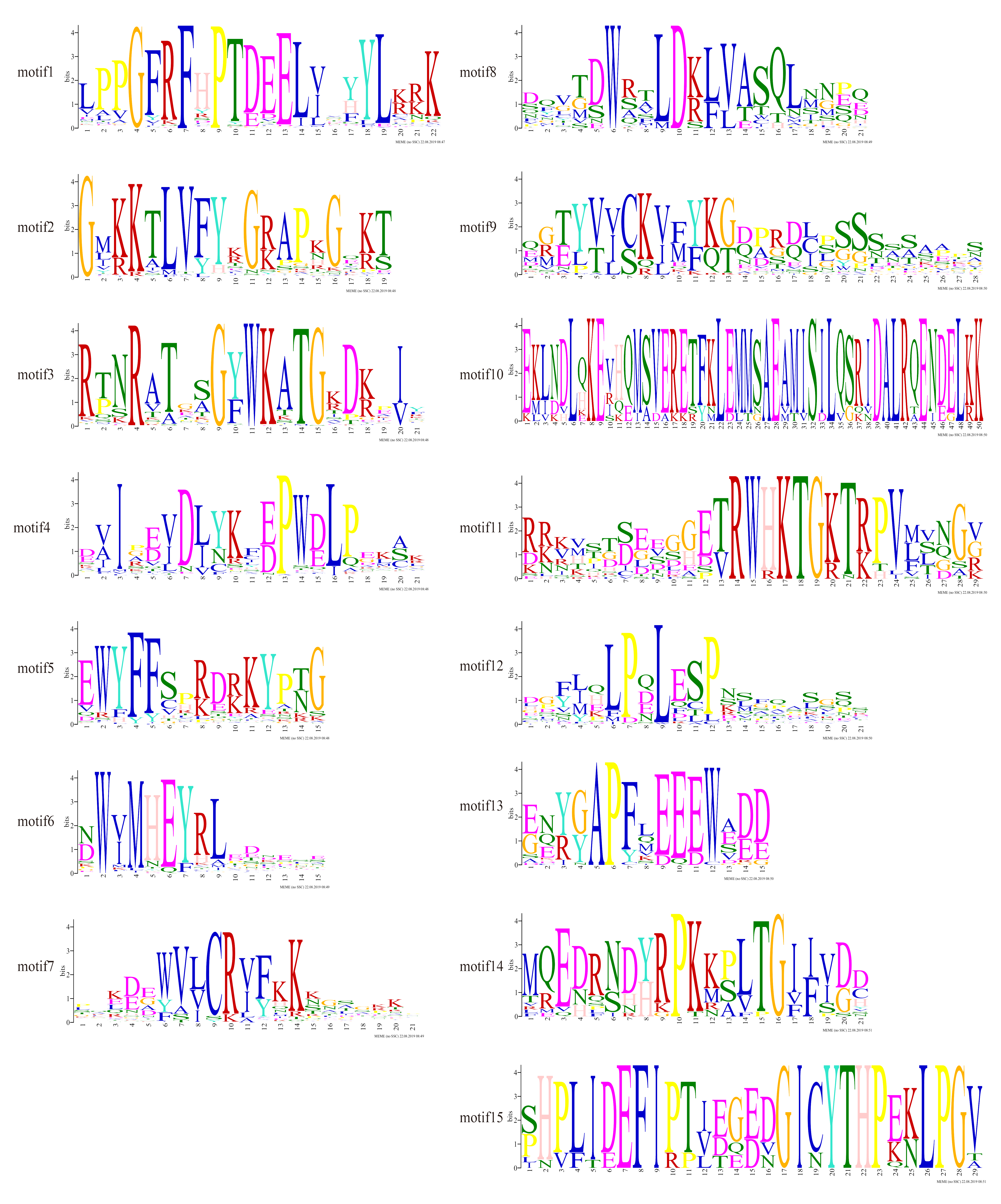

Supplement: Figure S4 — The height of each amino acid code in the sequence logo of each motif represents the degree of conservation. [file peerj-09-11212-s006.png]

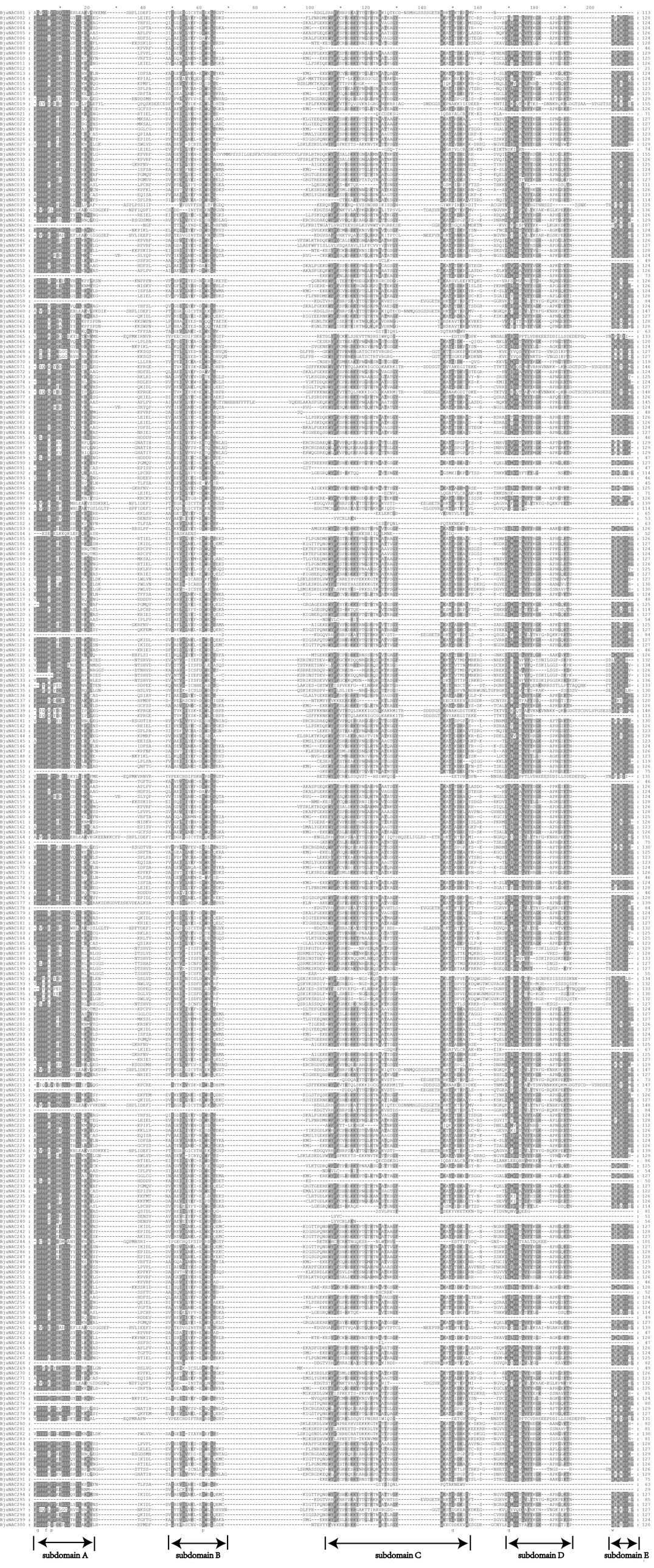

Supplement: Figure S5 — All domain sequences of BjuNACs divided into five subdomains A-E. [file peerj-09-11212-s007.png]

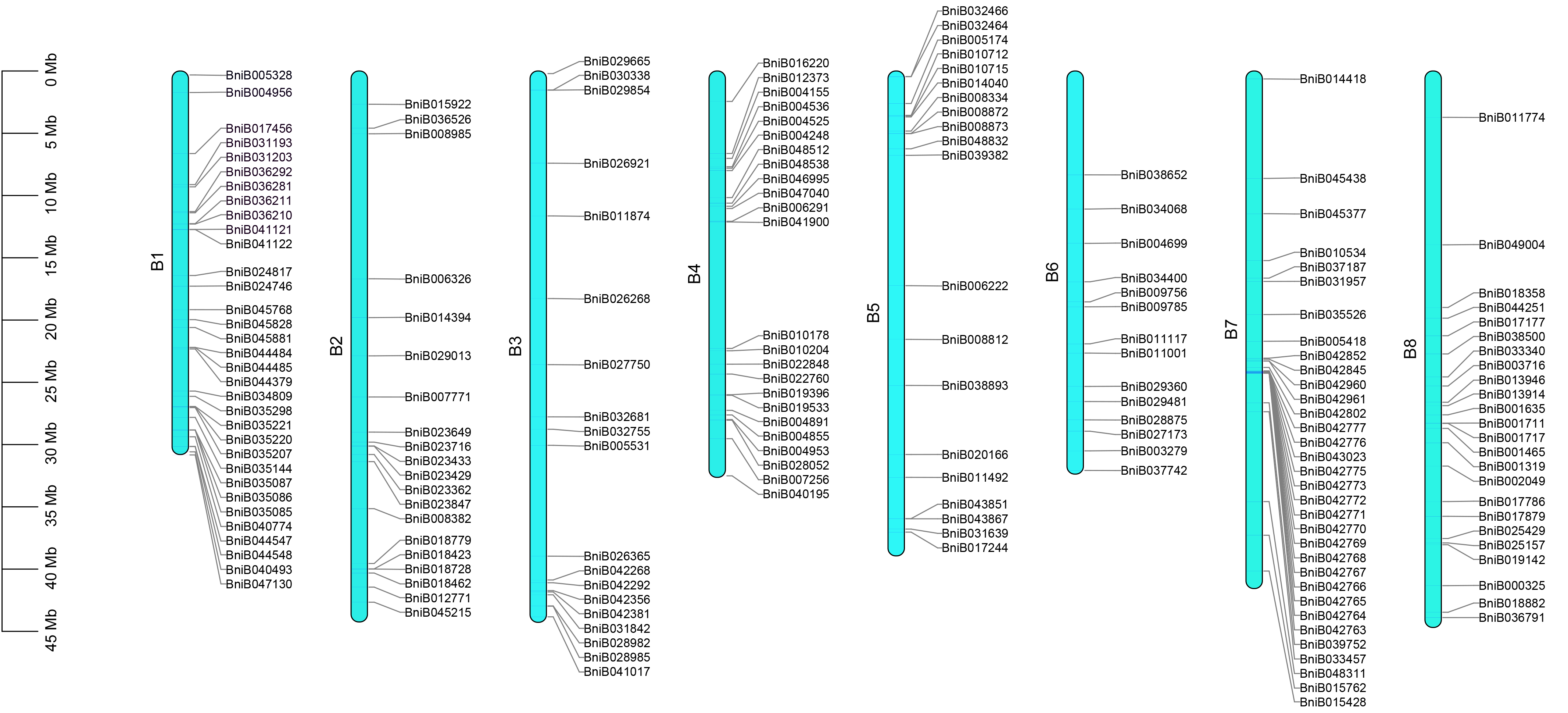

Supplement: Figure S7 — Graphical representation of locations for putative BniNAC genes on each chromosome. B1∼B8 represented the chromosome numbers. Other 25 BniNAC genes information belongs to scaffold data were shown in Table S3. [file peerj-09-11212-s009.png]
